# Spatial analysis by current multiplexed imaging technologies for the molecular characterisation of cancer tissues

**DOI:** 10.1038/s41416-024-02882-6

**Published:** 2024-10-22

**Authors:** Takashi Semba, Takatsugu Ishimoto

**Affiliations:** Division of Carcinogenesis, The Cancer Institute, Japanese Foundation for Cancer Research, Tokyo, Japan

**Keywords:** Cancer microenvironment

## Abstract

Tumours are composed of tumour cells and the surrounding tumour microenvironment (TME), and the molecular characterisation of the various elements of the TME and their interactions is essential for elucidating the mechanisms of tumour progression and developing better therapeutic strategies. Multiplex imaging is a technique that can quantify the expression of multiple protein markers on the same tissue section while maintaining spatial positioning, and this method has been rapidly developed in cancer research in recent years. Many multiplex imaging technologies and spatial analysis methods are emerging, and the elucidation of their principles and features is essential. In this review, we provide an overview of the latest multiplex imaging techniques by type of imaging and staining method and an introduction to image analysis methods, primarily focusing on spatial cellular properties, providing deeper insight into tumour organisation and spatial molecular biology in the TME.

## Introduction

Tumours are composed of various cellular and noncellular factors, including cancer cells, immune cells, fibroblasts, vascular endothelial cells, cytokines, and the extracellular matrix (ECM). Furthermore, each cell type is distinctly different in function, status, and differentiation, and this intracellular and intercellular heterogeneity creates a highly complex tumour microenvironment (TME). TME components play critical roles in tumour growth, metastasis, therapeutic resistance, and tumour suppression in a context-dependent manner by closely interacting with each other and ultimately affecting patient outcomes. Therefore, investigating the molecular characteristics of each TME component and their interactions is crucial to elucidate the mechanisms of tumour progression and to develop better treatment strategies.

The recent development of omics technologies has rapidly increased the resolution in cancer research from the bulk tumour level to the single-cell level. Single-cell RNA sequencing (scRNA-seq) methods, for example, have identified subgroups of tumour cells and the functional diversity of immune cells and stromal cells in the TME on the basis of their gene expression profiles [1]. For example, cancer-associated fibroblasts (CAFs) have been shown to play both pro- and antitumour roles over the past few decades [2]. ScRNA-seq has identified heterogeneous subtypes of CAFs with distinct functions (e.g., inflammatory cytokine secretion, ECM production, and antigen presentation) in many types of cancer [3–5], providing deeper insights into the complex roles of CAFs in the TME.

In addition to cell phenotypes, accumulating evidence suggests that the functional status of cells in the TME is strongly dependent on their cellular localisation and spatial relationships [6–9]. However, given that current standard single-cell sequencing technologies require tissue dissociation to obtain cell suspensions as input, the architectural and spatial information of the tumour will be inherently lost with these dissociative methods. Although spatially resolved transcriptomic methods have been developed recently and some methods have single-cell or subcellular resolution [10–13], implementation in routine laboratory experiments is impractical because of the limited accessibility of specialised instruments and the cost per run.

Formalin-fixed paraffin-embedded (FFPE) blocks are rich sources of clinical tissue with associated long-term clinical outcome information, and immunohistochemistry (IHC) is a widely used technique in cancer research and clinical diagnosis to determine protein expression in situ. In recent years, multiplex immunostaining techniques that can detect several to dozens of different antigen molecules on a single FFPE tissue section have been rapidly developed and have become popular in studies on clinical cohorts owing to their ease of application in clinical settings where IHC is routinely performed [14, 15].

In this review, we summarised the current multiplexed imaging technologies that have emerged as powerful tools in the TME research field over the past decade. We focused primarily on the technical aspects of staining, imaging and image analysis.

## Current multiplex imaging technologies

**Fig. 1:**
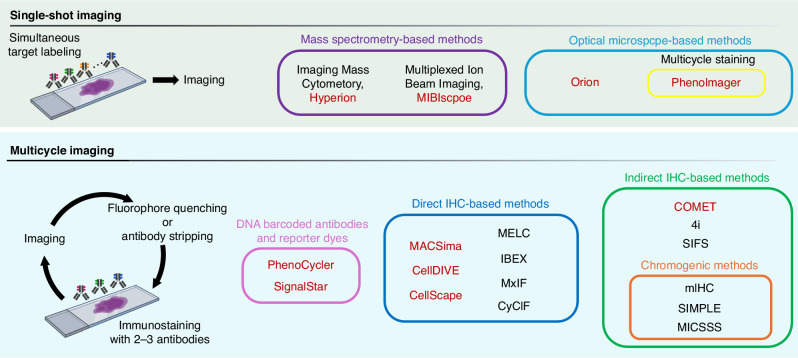
Overview of multiplex staining methods. Multiplex imaging technologies can be classified into single-shot (upper) and multicycle imaging approaches (lower). In single-shot imaging methods, targets are simultaneously stained with metal- or fluorophore-conjugated antibodies, except in Phenoimager, where the HRP-conjugated antibodies and fluorophore-labelled tyramide are sequentially stained and stripped to amplify the signal of target antigens, followed by imaging by specialised imaging equipment. Multicycle imaging methods are further divided on the basis of the antibody type, including DNA barcoded, fluorophore-conjugated, and unconjugated antibodies. Commercialised technologies are shown in red. The figures were created with BioRender.com.

We categorised current highly multiplex imaging technologies into two groups: single-shot and multicycle imaging approaches (Table 1 and Fig. 1). Although the principle of these methods for detecting proteins of interest is based on traditional IHC methods (e.g., antigen‒antibody reactions in situ), the readouts, required instruments, and antibody modifications vary based on the method used.

**Table 1:** Techniques and technologies for multiplex imaging

Method/Platform name	Approach	Antibody type	Antibody stripping or fluorophore bleaching method/Company	Number of targets	Reference
MIBI/ MIBIscope	Mass spectrometry-based single-shot imaging	Metal-conjugated primary antibody	Ionpath	40	[16, 17]
IMC/ Hyperion	Mass spectrometry-based single-shot imaging	Metal-conjugated primary antibody	Standard BioTools	40	[18]
Orion	Fluorescence microscope-based single-shot imaging	Fluorophore-conjugated primary antibody	RareCyte	20	[22]
PhenoImager	Fluorescence microscope-based single-shot imaging	HRP-conjugated primary antibody and TSA technology	Microwave treatment	7	[21]
PhenoCycler	Oligo-barcoded antibody-based multicycle imaging	DNA barcoded primary antibody and fluorescent dye-conjugated oligonucleotide	Chemical stripping of fluorescent oligonucleotide	50	[27, 28]
SignalStar	Oligo-barcoded antibody-based multicycle imaging	DNA barcoded primary antibody and fluorescent dye-conjugated oligonucleotide	Chemical stripping of fluorescent oligonucleotide	8	[29]
MxIF	Direct IHC-based multicycle imaging	Fluorophore-conjugated primary antibody	Alkaline H_2_O_2_ chemistry (US patent 7741045)	61	[32]
CyCIF	Direct IHC-based multicycle imaging	Fluorophore-conjugated primary antibody	4.5% H_2_O_2_ and 24 mM NaOH in PBS	60	[33]
IBEX	Direct IHC-based multicycle imaging	Fluorophore-conjugated primary antibody	1 mg/ml solutions of LiBH4	65	[34]
MELC	Direct IHC-based multicycle imaging	Fluorophore-conjugated primary antibody	Soft photobleaching	90	[31]
MACSima	Direct IHC-based multicycle imaging	Fluorophore-conjugated primary antibody	Miltenyi Biotech	60	[39]
Cell DIVE	Direct IHC-based multicycle imaging	Fluorophore-conjugated primary antibody	Leica Microsystems	60	[40]
CellScape	Direct IHC-based multicycle imaging	Fluorophore-conjugated primary antibody	Canopy Biosciences	18	[41]
COMET	Indirect IHC-based multicycle imaging	Off-the-shelf primary antibody	Lunaphore	40	[42]
4i	Indirect IHC-based multicycle imaging	Primary antibody + fluorophore-conjugated second antibody	Elution buffer consists of 0.5 M L-glycine, 3 M urea, 3 M guanidinium chloride, and 70 mM TCEP- HCl in ddH2O adjusted to pH 2.5	40	[38]
SIFS	Indirect IHC-based multicycle imaging	Primary antibody + fluorophore-conjugated second antibody	Glycine buffer, pH 2.0 (Elution) followed by microwave cooking for 2 to 5 min in a 0.1 M citrate buffer, pH 6.0 (denaturation)	6	[35]
MICSSS	Indirect IHC-based multicycle imaging	Primary antibody + HRP-conjugated secondary antibody (Chromogenic, AEC)	Organic solvent (50% ethanol, 2 min; 100% ethanol, 2 min; 100% xylene; 2 min, 100% ethanol, 2 min; and 50% ethanol, 2 min) + heating	10	[43]
mIHC	Indirect IHC-based multicycle imaging	Primary antibody + HRP-conjugated secondary antibody (Chromogenic, AEC)	Destaining with ethanol and microwave heat with antigen retrieval citrate solution	29	[37]
SIMPLE	Indirect IHC-based multicycle imaging	Primary antibody + HRP-conjugated secondary antibody (Chromogenic, AEC)	Destaining with ethanol and antibody elution with acidified KMnO4	12	[36]

### Single-shot imaging methods

#### Mass spectrometry-based approaches

There are two major mass spectrometry-based imaging methods that use heavy metal-tagged antibodies: multiplexed ion beam imaging (MIBI) [16, 17] and imaging mass cytometry (IMC) [18]. Both methods can detect more than 40 targets via one-shot staining of typical 4–7 µm thick tissue samples with an antibody cocktail and one-shot signal acquisition via ionisation of antibody-linked metal isotopes followed by quantification via time‒of–flight (TOF) mass spectrometry. For MIBI, an oxygen duoplasmatron primary ion beam is utilised to release secondary ions from both metal elements and tissue-endogenous elements, whereas for IMC, an ultraviolet laser is used to ablate tissue, and the aerosolized tissue is ionised with plasma. The maximum resolution of each method is 0.4 µm per pixel in the MIBIscope and 1 µm per pixel in the IMC. A detailed comparison between MIBI and IMC has been presented in other recent reviews [19, 20]. Instruments for both the MIBI and IMC methods have been commercialised as MIBIScope from IONpath and Hyperion from Standard BioTools, respectively. Ready-to-use metal-labelled antibodies are available from some suppliers, and metal conjugation kits for in-house antibody labelling are also commercially available. The use of nonbiological metal isotopes allows researchers to disregard background signals, which is a critical advantage of mass spectrometry-based imaging methods. Owing to the single cycle of staining and imaging, tissue damage is minimised compared with that of multicycle imaging methods. However, given that laboratories equipped with specialised instruments are limited, mass spectrometry-based imaging is not yet suitable for daily practical use.

#### Fluorescence microscope-based approaches

Another one-shot imaging approach is multiplex immunofluorescence using multispectral imaging platforms. The PhenoImager HT from Akoya Biosciences (formerly the PerkinElmer Vectra Polaris) visualises five to six targets based on fluorescent signals enhanced by tyramide signal amplification technology [21]. In this method, a slide is stained with an unconjugated primary antibody and a horseradish peroxidase (HRP)-bound secondary antibody, followed by incubation with fluorophore-labelled tyramide. HRP-catalysed tyramide covalently binds tyrosine residues on or near the target protein, depositing the site of the antigen of interest. After signal amplification, the antibody complex is stripped by microwave treatment, and this staining cycle is repeated to generate up to seven-colour slides. The Orion platform from RareCyte recently achieved whole-slide 16- to 18-plexed immunofluorescence imaging at 0.325 µm per pixel resolution via seven excitation lasers assembled with a tunable emission filter and computational unmixing of individual fluorophore signals [22]. The samples are stained with a mixture of antibodies conjugated with spectrally separated fluorophores at the same time, reducing the time required for sample preparation. These methods have a faster scanning speed, higher resolution, and larger imaging area than those of mass spectrometry-based imaging. However, these methods also require specialised imaging devices and dedicated optimised fluorophores to label antibodies.

### Multicycle imaging methods

For the optical microscope-based approach, spectral overlap is one of the main impediments to achieving highly multiplexed imaging. In recent decades, many methods have been developed to overcome this issue based on repetitive imaging of a single target by chromogenic detection or a limited number of targets with spectrally distinct fluor dyes [23–26]. The common procedure of these methods involves repeating cycles consisting of the following steps: (1) immunostaining with target-specific antibodies; (2) image acquisition by detecting either chromogenic or fluorescence signals; and (3) destaining. These methods can be further grouped into subgroups on the basis of antibody modifications, such as oligonucleotide-labelled antibodies or fluorophore-labelled antibodies.

#### Oligo-barcoded antibody approaches

A typical example of an approach using oligonucleotide-labelled antibodies is the PhenoCycler system from Akoya Biosciences (formally known as CODEX) [27, 28]. In this system, samples can be simultaneously stained with oligonucleotide-barcoded antibodies against more than 50 targets. After staining, the targets are detected via iterative cycles of hybridisation of three fluorescent dye-tagged complementary oligonucleotides (reporters), imaging, and removing the reporters. Simultaneous antibody staining and the automated cycles of hybridisation, imaging, and dehybridization increase throughput and tissue integrity. Oligonucleotide-conjugated antibodies are commercially available from predesigned panels or can be prepared via a custom conjugation kit. SignalStar [29] from Cell Signalling Technology is another oligonucleotide-based multiplex IHC system that has recently become commercially available and enables the detection of up to 8 targets in FFPE tissue by manual staining or with an RX BOND automated IHC staining system (Leica).

#### Direct and indirect IHC approaches

For signal detection via IHC, the most common method in research laboratories is the use of an antibody conjugated to either an enzyme, such as HRP, or a fluorophore for chromogenic IHC or fluorescent IHC. The number of antigens that can be detected on a slide is normally one to three in routine IHC due to the limited colours or fluorescent dyes available for ordinary brightfield or fluorescence microscopy. The host species of the antibodies further confounds the situation, especially with the indirect IHC method. In 1968, Nakane reported that antibodies bound to antigens on slides can be eluted with low-pH glycine–hydrochloride acid buffer and demonstrated that three antigens can be detected on the same slide via indirect IHC with HRP-labelled secondary antibodies and different colour substrates [30]. Since then, numerous methods for such sequential immunostaining have been reported and can be classified into two groups: direct IHC with fluorophore-conjugated primary antibodies followed by quenching of fluorescent signals [31–34] or indirect IHC with fluorophore- or enzyme-conjugated secondary antibodies followed by denaturing the antibodies. [23–25, 35–38] Although tissue degradation through cycles of staining and destaining is a concern and the procedure is relatively labour-intensive, antibody-based multicycle imaging is the approach with the lowest barrier to entry because the microscope and the antibody resources currently available in the laboratory can be utilised with only minimal modification of the protocol.

Multiepitope-ligand cartography (MELC [31]), multiplexed fluorescence microscopy (MxIF [32]), tissue-based cyclic immunofluorescence (t-CyCIF [33]), and iterative bleaching extends multiplexity (IBEX [34]) are representative methods of direct IHC-based multicyclic imaging. The main difference among these methods is how the fluorescent inactivation is performed. For MELC, a photobleaching approach is used to remove fluorescein isothiocyanate (FITC) or phycoerythrin (PE) signals. MxIF uses a chemical bleaching approach in an alkaline solution containing hydrogen peroxide and effectively inactivates cyanin-based fluorescence. In the t-CyCIF method, a combination of chemicals (hydrogen peroxide, high pH) and photobleaching is utilised to eliminate various fluorophores. For IBEX, lithium borohydride (LiBH_4_) is used for fluorophore quenching. While LiBH_4_ was found to be capable of bleaching most fluorescent dyes, Alexa Fluor 594 and some nuclear dyes, such as JOJO-1 and Hoechst, were resistant to bleaching. These bleaching-resistant dyes, however, were found to be helpful as fiducials for image registration. Automated staining and imaging platforms based on such direct IHC approaches are now commercially available from several companies, including MACSima [39] from Miltenyi Biotech, Cell DIVE [40] from Leica Microsystems, and CellScape [41] from Canopy Biosciences.

**Fig. 2:**
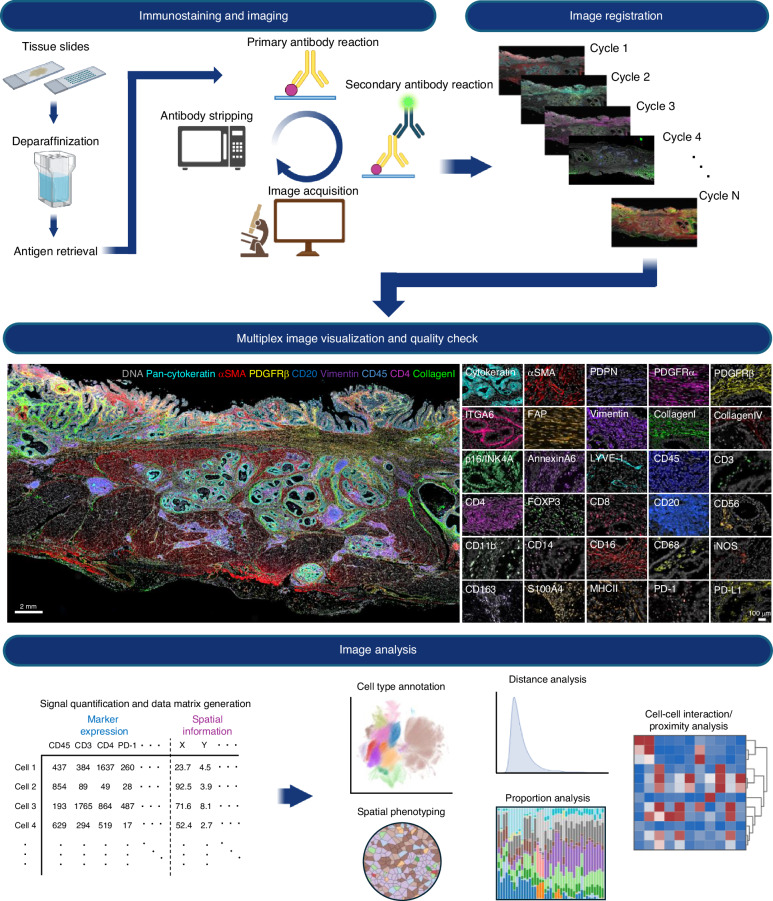
An example of a multiplex imaging workflow. An example of the workflow of indirect IHC-based multicycle imaging adapted in our laboratory. In the first round, routine indirect IHC methods, such as deparaffinization, antigen retrieval, blocking, staining, and mounting, can be performed as usual. Notably, during the initial imaging, the area’s location to be imaged (x, y coordinates, etc.) should be recorded, as the same area should be imaged in future cycles. We used a VS120 (Olympus) slide scanner capable of acquiring WSIs. After imaging, the coverslip was carefully removed, and the slide was washed in a buffer such as PBS. Antibody stripping was then performed by microwave treatment in Tris-EDTA buffer at pH 9, and the slides were subjected to the next cycle. After completion of the staining and imaging cycle, the raw image formats, such as .vsi, were converted into the OME-TIFF format via QuPath. The converted OME-TIFF images of each cycle were then registered into one multichannel image via the palom or wsireg package. A quality check of the image was performed after registration to ensure that the registration was correct. The MCMICRO pipeline was used for cell segmentation and signal quantification of images or regions of interest. After obtaining a single-cell feature matrix, which includes the expression of each marker protein and spatial information, we performed dimensionality reduction and clustering, followed by cell phenotyping, in a manner similar to that used for scRNA-seq data analysis. The map of phenotyped cells was then overlaid on the original image to assess the concordance between the phenotype and the marker expression pattern, and various spatial features, such as the cell proportion, cell‒cell distance and communication, were analysed. The figures were created with BioRender.com.

Indirect IHC approaches have the key advantage of an enhanced signal, as multiple secondary antibodies can bind to the primary antibody, increasing the sensitivity to low-expression antigens. In addition, the use of unconjugated primary antibodies increases the availability of antibodies and the flexibility of the experimental design. In the early 2000s, Wählby et al. developed a protocol for sequential immunofluorescence staining (SIFS [35]), in which stained tissue slides were incubated with low-pH glycine buffer followed by microwave treatment, and the primary and secondary antibodies as well as the fluorophores were removed. For iterative indirect immunofluorescence imaging (4i [38]), the primary and secondary antibodies were removed with elution buffer consisting of glycine, urea, guanidinium chloride, and tris(2-carboxyethyl) phosphine hydrochloride, and up to 40-plex targets were detected by spinning-disk confocal microscopy at the subcellular scale. Notably, cultured cell samples, not FFPE samples, were used in the 4i method. Lunaphore offers the COMET [42] instrument, which automatically repeats cycles of immunostaining, imaging, and antibody elution, working with off-the-shelf primary antibodies and fluorescently labelled secondary antibodies. An example workflow of the indirect IHC approach is depicted in Fig. 2.

The chromogenic indirect IHC approach has also been used for multicycle imaging methods, such as sequential immunoperoxidase labelling and erasing (SIMPLE [36]), multiplexed consecutive immunohistochemical staining on single slides (MICSSS [43]), and multiplexed immunohistochemistry (mIHC [37]). These methods rely on 3-amino-9-ethyl carbazole (AEC), an ethanol-soluble chromogenic substrate of HRP, which can be destained by ethanol washing, and SIMPLE utilises acidified KMnO_4_ buffer, whereas MICSSS and mIHC use heat treatment in an acidic or basic buffer for antibody elution. Although the use of a conventional brightfield microscope and chromogenic IHC is amenable to clinical application, the number of targets to be detected in a single cycle is inherently limited (i.e., one target per cycle), and achieving a high-plex image is challenging.

## Image analysis

As with recent large-scale data, such as transcriptomic or genomic data, multiplex image analysis requires several steps of computational data processing: image registration, cell segmentation, quantification of protein expression, normalisation, cell phenotyping, and spatial analysis. This section provides an overview of each analysis and the software used to perform them, focusing on more versatile open-source software, although applications for such image analysis are available on the market from several companies.

### Image registration

When a whole-slide image (WSI) is acquired, most imaging devices divide the entire image into tiled images of several hundred pixels per slide and then assemble the tiles into an image file (stitching) to capture a large area while maintaining high resolution. While whole-slide scanners are usually equipped with specialised software to stitch the acquired image tiles automatically, if necessary, tiled images can also be stitched by open-source stitching software such as BigStitcher [44]. Along with stitching, some illumination corrections can be performed by software, such as BaSiC [45] and SSCOR [46], to correct the shading, strips or artefacts in the stitched fluorescence images. For multicycle imaging, the images acquired in each cycle must be precisely aligned at pixel resolution and superimposed (registration) because of sample movement between the imaging rounds. Several open-source programmes are available to achieve image registration, such as Alignment by Simultaneous Harmonisation of Layer/Adjacency Registration (ASHLAR) [47], elastix [48], piecewise alignment for layers of mosaics (palom) (https://github.com/labsyspharm/palom), and wsireg (https://github.com/NHPatterson/wsireg). ASHLAR uses the tiled images obtained in each cycle as input for stitching and registration, whereas other software performs registration of WSIs that have already been stitched by other software. Each cycle of images requires a common fiducial marker channel, typically a nucleus channel stained with a DNA dye, such as 4′,6-diamidino-2-phenylindole (DAPI).

### Cell segmentation and quantification of signals

Cell segmentation is the process of separating individual cell regions from other regions in an acquired image. Many software programmes can be used for cell segmentation, including Fiji [49], ilastik [50], CellProfiler 4 [51], Cytokit [52], UnMiCST [53], Steinbock [54], Cellpose [55], and MESMER [56]. MESMER uses both nuclear and cytoplasm images as input to improve the accuracy of segmentation. Most cell segmentation methods adopt deep learning approaches, mainly semantic segmentation (e.g., pyramid scene parsing networks [57] and UNet-based networks [58]) and instance segmentation (e.g., Mask R-CNN [59]). Bankhead et al. developed QuPath, an open-space software for interactive WSI analysis [60]. QuPath can read images, particularly very large 2D images obtained from whole-slide scanners in a wide range of file formats, and seamlessly perform cell segmentation for expression analysis. Owing to the Bio-Formats library (https://www.openmicroscopy.org/bio-formats/), QuPath also supports the conversion of each company’s proprietary file format (e.g., .czi, .vsi, and .ndpi) into a highly versatile format, such as TIFF or OME-TIFF. Squidpy is a Python-based framework for the analysis and visualisation of spatial omics data, from cell segmentation to signal quantification and spatial analysis [61]. imcRtools is a similar integrated analysis software based on R [54]. Multiple Choice MICROscopy (MCMICRO [62]) is a suite of software packages for image processing, cell segmentation, and quantification in Docker/Apptainer (formerly Singularity) containers, and the pipeline is implemented in NextFlow [63], which can be run on many computer platforms, or Galaxy [64], a web-based interface for running analysis platforms. MCMICRO also allows the visualisation of multiplex images and the results of analyses via Minerva, a suite of tools for the interactive viewing of whole-slide multiplex images on the web [65]. More information on cell segmentation and analysis platforms can be found in other reviews [66, 67].

### Image normalisation

Image normalisation is the process of adjusting the intensity levels of images to make the pixel values or fluorescence intensities from images acquired from different times, sites, or batches comparable. Although there is still no consensus, image normalisation methods in multiplex immunohistochemistry (IHC) have advanced actively, focusing on improving accuracy by addressing sample variability, noise, and artefacts. For example, Robust intEnSiTy nOrmalization mEthod (RESTORE) [68] uses putative mutually exclusive marker pairs, such as CD45 and cytokeratin, and infers autofluorescence levels to normalise raw fluorescence intensities. Graf et al. proposed the immunoFLuorescence Image normalisation (FLINO) [69] method, where the nuclear dye DAPI was used as a “ground truth”, and found that several normalisation methods, such as upper quantile normalisation, median ratio normalisation [70], and trimmed mean of the M values [71], applied to unbiased grid objects in log space were robust to the presence of outliers and experimental artefacts. Harris et al. compared several transformation and normalisation methods and reported that the mean division and mean division with log transformation methods are ‘good enough’ in practice, as they are simple and computationally efficient [65]. Software packages for image normalisation, such as mxnorm [72], have also been developed recently, which allows users to test several algorithms.

### Cell phenotyping

Cell phenotyping is a fundamental and crucial step in multiplex image analysis. Typically, several to dozens of cell types (e.g., immune cells, tumour cells, and stromal cells) must be identified via multiplex image analysis based on the expression of markers specific to each cell type. In addition, determination of the phenotype or functional status of cells within the same cell type (e.g., CAF subtypes or CD8+ T-cell exhaustion status) is often necessary.

For multiplex images with information on many markers, unsupervised clustering or marker gating strategies are generally applied for cell phenotyping. In unsupervised clustering methods, such as the k-means [73], Louvain [74], and Leiden [75] algorithms, each cell is grouped on the basis of marker expression information without predefined thresholds. K-means is a distance-based clustering algorithm that divides data into a predefined number of clusters, whereas Louvain and Leiden are graph-based methods used primarily for community detection, particularly for network data, and it is not necessary to specify the number of clusters beforehand. These algorithms are widely implemented in software, such as Phenograph [76], Seurat [77], Scanpy [78], and Giotto [79].

The marker gating strategy is commonly used not only in cytometry but also in multiplex image analysis. The expression levels of markers in cells are plotted, typically as 2D scatter plots, and gates are manually drawn on the scatter plots to separate cell populations on the basis of marker intensity. Software, such as histoCAT [80] and Cellprofiler Analyst [81], can be used for such marker gating. Nirmal et al. developed a gating-based phenotyping approach that can automatically and unsubtly annotate clusters via a relationship chart based on cell type and marker expression information and was implemented in SCIMAP software [82, 83]. In this approach, the expression level of each marker is first rescaled from 0 to 1, and a cut-off of 0.5 is used to determine whether the cell expresses the marker. The cells are then assigned to a predefined cell type according to the relationship chart based on the expression pattern of that marker.

The unsupervised clustering method can handle large datasets with numerous markers and has the potential to discover unexpected and unknown cell types or cell statuses. However, the annotation of clusters is often time-consuming because the clustering results can be difficult to interpret, especially when clusters are not well separated or when the relationship between the markers and their biological significance is unclear. The advantages of the marker gating strategy are that it is straightforward and visually interpretable, and it will be helpful when identifying well-characterised cell types. However, gating becomes difficult with a large number of markers since handling many variables increases the complexity.

Regardless of the method used, the experimenter should always review the images with their own eyes after phenotyping.

#### Spatial analysis

**Fig. 3:**
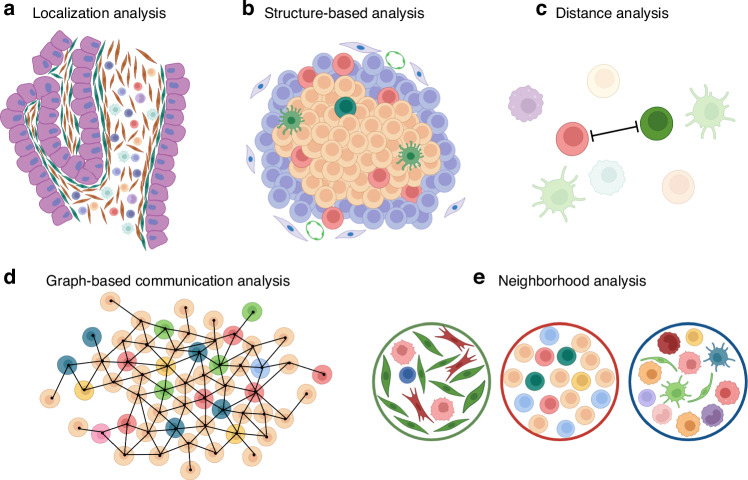
Overview of the spatial analysis of multiplex images. Analyses were based on the spatial location of each cell after cell phenotyping. **a** Localisation analysis that determines where certain cell types are distributed within a tumour or what cell types are distributed in specific locations. **b** Structure-based analysis that focuses on certain structures, such as TILs and blood vessels, within the tumour tissue. **c** Analysis based on the distances between cells. **d** Graph-based cellular communication analysis to investigate communication between cells via mathematical algorithms. **e** Neighbourhood analysis based on the composition of cell populations repeatedly observed within a specific range. The figures were created with BioRender.com.

After cell annotation has been completed, several analyses based on the spatial location of each cell type need to be performed (Fig. 3). Here, we outlined the current spatial analysis by dividing it into (1) analysis based on the spatial distribution of cell types, (2) analysis focused on the structure within the tissue, (3) analysis of distances between cells, (4) analysis of communication between cells based on mathematical algorithms, and (5) analysis based on the composition of cell populations that are repeatedly observed within a specific range.

### Localisation analysis

The analysis of where certain cell types are distributed within a tumour or what cell types are distributed in specific locations is critical for determining the nature of a cancer. This type of analysis is frequently performed to determine where immune cells, such as CD8 T cells, are distributed within or outside the tumour [84] (Fig. 3a). We recently identified mesothelial cells of the peritoneum that underwent mesothelial–mesenchymal transition (MMT) in the tumour stroma via multiplex imaging [85]. In peritoneally disseminated lesions from gastric cancer patients, calretinin-positive mesothelial cells were observed in the stroma legion adjacent to EpCAM- and syndecan-1-expressing tumour cells, which also expressed the mesenchymal markers alpha-smooth muscle actin (SMA) and tenascin-C, suggesting that they were mesothelial cells that had undergone MMT. The tenascin-C produced by such MMT-induced mesothelial cells is known to interact with syndecan-1 in tumour cells, and experiments using tenascin-C knockout mice and syndecan-1 knockout tumour cells have further shown that these interactions play essential roles in the dissemination of gastric cancer [85]. Grout et al. identified four major subtypes of CAFs in non-small cell lung carcinoma (NSCLC) via scRNA-seq analysis and confirmed these CAFs by multiplex imaging. These researchers reported that most CAFs observed in early NSCLC tissues were alcohol dehydrogenase 1B (ADH1B)-positive CAFs or MYH11-alpha-SMA-positive CAFs, whereas in advanced stages, fibroblast-activating protein (FAP)-positive CAFs and FAP-positive alpha-SMA-positive CAFs predominated in advanced stages, among which MYH11-positive alpha-SMA-positive CAFs and FAP-positive alpha-SMA-positive CAFs were thought to contribute to immune cell exclusion through their location at the lining of the tumour nest.

### Structure-based analysis

The tumour is not a disorganised collection of cells but is composed of various structures that are deeply involved in tumour progression and control (Fig. 3b). Recent studies revealed that the presence of B cells and tertiary lymphoid structures (TILs) in tumours was associated with the response to immune checkpoint blockade (ICB) therapy [86, 87]. Helmink et al. demonstrated the detailed architectural characteristics of TILs in melanoma and other cancer types via multiplex imaging [86]. They reported that CD20-positive B cells were localised in the TILs of tumours from ICB responders and were colocalized with CD4 T cells, CD8 T cells, FOXP3-positive regulatory T cells (Tregs), and CD21-positive follicular dendritic cells, suggesting the presence of mature secondary follicle-like TLSs. They also presented increased activation markers on T cells inside TILs than on those outside TILs, suggesting that B cells in TILs contribute to T-cell activation and the antitumour response. Hua et al. reported that vascular endothelial cells within tumours were transformed into MECA79-positive high-endothelial venules (HEVs) via combination therapy with anti-vascular endothelial growth factor receptor 2 and immune checkpoint blockade (ICB) in a mouse model [88]. Multiplex imaging analysis revealed a high frequency of progenitor CD8 T cells near HEVs in such tumours, suggesting that HEVs create a supportive environment in which progenitor CD8 T cells self-renew and expand into cytotoxic T cells [88]. These analyses focusing on macrostructures within tumours are expected to continue to contribute to the discovery of clinical prognostic markers, and multiplex imaging techniques can be powerful tools to uncover the details and heterogeneity of these structures that cannot be revealed by H&E staining or conventional few-colour IHC.

### Cell–cell distance

The distance between cells is essential for determining functional cell‒cell interactions (Fig. 3c). In non-small cell lung cancer, a shorter distance between CD8 effector T cells and pan cytokeratin-positive tumour cells was reported to correlate with longer survival, whereas a shorter distance between CD163-positive M2 macrophages and M1 macrophages was strongly associated with poor prognosis [89]. Phillips et al. reported that in cutaneous T-cell lymphomas, multiplex imaging analysis using CODEX revealed no difference in the infiltration frequency of CD4 T cells or CD8 T cells between patients who responded to ICB and those who did not, whereas differences were found in the spatial distance relationships between PD-1-positive CD4 T cells, Tregs, and tumour cells [90]. They defined the ratio of these distances between immune cells and tumour cells as the *SpatialScore*, and a low *SpatialScore* (i.e., PD-1-positive CD4 T cells are closer to tumour cells) is correlated with increased T-cell effector activity, and a high *SpatialScore* (i.e., PD-1-positive CD4 T cells are closer to Tregs) is correlated with increased T-cell suppressive activity. In fact, the *SpatialScore* of patients who responded to ICB was markedly lower than that of patients who did not respond [90].

### Cellular communication via a graph-based approach

In addition to being based on mere cell-to-cell distances, mathematical methods such as those used in geospatial information analysis can be adopted to reveal spatial cell‒cell interactions (Fig. 3d). Jackson et al. used IMC multiplex imaging to analyse cellular communities within tumour tissue in patients with breast cancer using a graph-based approach with the Louvain method to identify communities of neighbouring cells, which were then used to stratify patients and find correlations with their prognosis [91]. Gaglia et al. used Delaunay triangulation in a mouse model of lung cancer to identify a cellular network of intratumoral lymphocytes called “lymphonets” [9]. These researchers developed a pair of mouse models: a Cre recombinase-mediated Kras/Trp53 mutant mouse model (KP) and a mouse model overexpressing an antigenic peptide to increase immunogenicity (KP-LucOS). Using CyCIF-based multiplex imaging, these researchers revealed that in the KP-LucOS model, lymphonets were more frequently present inside tumours, and the proportion of CD8+ T cells in the lymphonet was greater, whereas the proportion of Tregs was lower [9].

### Neighbourhood analysis

In addition to analyses of the distance or communication among specific cell types, the pattern of the composition of the cell types surrounding each tumour cell or other cell type has recently been utilised to characterise the tumour phenotype and clinical outcome (Fig. 3e). In colorectal cancer, Schurch et al. identified patterns of the cellular neighbourhood (CN) with a similar composition of cell types, which are conserved among patients, and these CNs were correlated with distinct immune TMEs and patient survival [28]. Another group proposed an approach to define neighbouring cells within a certain (20–50 µm) radius from the centre of the cell as its CN [9, 82]. For example, ten recurrently observed CNs [recurrent cellular neighbourhoods (RCNs)] with distinct cellular compositions were identified in melanoma tissue and were associated with tumour progression. RCN1, which is composed mainly of keratinocytes and Langerhans cells, was detected in the epidermis, whereas RCN10, which is composed of SOX10-positive melanoma cells, was detected in regions of vertical growth phase melanoma. The frequency of immune cell-enriched RCNs also differs between precursor and progressive RCNs, suggesting increased complexity of the immune environment [82].

## Perspective

The technical aspects of multiplex imaging and image analysis have been outlined thus far. New technologies, methods, and analytical techniques continue to be introduced, and the field is rapidly developing. The issues to be overcome and the expected developments in multiplex imaging in the future need to be divided into clinical and basic research aspects.

First, one of the most important clinical applications of multiplex imaging may be in the field of biomarker development. Currently, a single marker is often used to determine the predictive value of ICB and other cancer therapies (e.g., IHC of PD-L1 in anti-PD-1/PD-L1 therapy), but this method is insufficient for treatment decisions [92, 93]. One potential direction is to use multiplex imaging techniques to detect combinations of multiple molecules at once, which would increase the accuracy of the prediction of therapeutic efficacy or patient prognosis. For example, Zugazagoitia et al. demonstrated that CD56 and CD4 counts measured in the CD45-positive compartment by multiplex imaging can be used as markers for predicting good clinical outcomes of NSCLC patients treated with immunotherapy [94]. This group further demonstrated that CD66b expression in CD45- and CD68-positive immune stroma was associated with shorter survival in immunotherapy-treated NSCLC patients [95]. Another direction is to use spatial analysis for biomarker discovery. As noted in the previous section, analysis of the positional relationship between cells and the cell types near a particular cell can provide a better understanding of the actual state of the TME than analysis of the number or frequency of each cell. Chen et al. established a signature of immune cell infiltration for gastric cancer based on the density of subsets of CD4, CD8 T cells and CD68-positive macrophages and the spatial organisation of PD-1-positive LAG3-negative CD8 T cells. This signature can predict the response to anti-PD-1/PD-L1 therapy and the survival of patients with gastric cancer.

To ensure the relevance of such biomarker development, standardisation of the imaging, analysis, and reporting methods is extremely important. As discussed above, the technologies, equipment, and antibodies for multiplex imaging have been developed by different groups and have then evolved independently. From a technical point of view, a single-shot imaging approach that detects many targets at once would be most suitable since the current multicycle imaging methods of repetitive staining and imaging cycles often cause tissue loss and technical variance among experimenters and institutions. Currently, image analysis methods, including survival analysis, also vary from study to study. For survival analysis, whether to use the number of positive cells or expression intensity for a given biomarker or whether to use the median or quartile as the cut-off value has not been determined. On the other hand, regarding the reporting method, the minimum information guidelines for highly multiplexed tissue images (MITI) have been proposed recently as guidelines for reporting information on multiplex IHC images [96]. MITI focuses on metadata such as clinical metadata, biospecimen metadata, and image metadata, including microscopes, acquisition parameters, channels, and analyses. The Human Tumour Atlas Network (HTAN) [97], which is the National Cancer Institute-supported database that integrates various types of tissue imaging and molecular data of tumours, is based on MITI.

To minimise variations in technical and analytical methods, it will eventually be necessary to conduct multicentre clinical trials to standardise the methods. In addition, since diagnostic imaging with the help of artificial intelligence (AI) has already been implemented in clinical practice, the use of AI and machine learning in the field of multiplex image analysis, where development is very active, is expected to lead to more reproducible analysis among observers and institutions.

In contrast, in basic research, integration with other omics methods, such as genomics, epigenomics, transcriptomics, and metabolomics, is rapidly progressing to gain deeper insight into the TME [98]. For example, recent developments in spatial transcriptomics technologies, such as the Xenium platform from 10X Genomics [13], achieved subcellular resolution with hundreds to thousands of gene expression profiles and enabled more analytic approaches, such as spatial trajectory analysis and cell‒cell communication analysis [99]. The CosMX platform from NanoString also enables the simultaneous detection of more than one thousand genes and 64 protein markers [100], which can complement the comprehensiveness of multiplex imaging. Takei et al. developed a method of sequential fluorescence in situ hybridisation (seqFISH) for RNA and DNA together with sequential immunofluorescence, which can visualise thousands of genes and transcriptionally active gene sites, along with chromatin marks of cultured cells at subcellular resolution [101, 102]. Currently, these researchers have expanded their method to tissue-based multiomic imaging, achieving simultaneous mapping of more than 100,000 genomic loci and transcriptomes for more than 17,000 genes and 27 markers of subnuclear structures [103]. Hu et al. proposed the single-cell spatially resolved metabolic (scSpaMet) framework, which combines TOF secondary ion mass spectrometry and the IMC method, to visualise more than 200 metabolic markers and 25 protein markers in the same tissue [104]. Using the scSpaMet pipeline, these researchers revealed distinct metabolic statuses in the tumour and stromal areas and metabolic competition between tumour cells and immune cells. Further technological innovations in the future are expected to expand the analytic area and increase the resolution of these spatial multiomic analyses.

Three-dimensional (3D) analysis is another actively developing and exciting field of multiplex imaging. Several groups have performed multiplex imaging via the IMC or CyCIF methods on numerous serial sections and reconstructed the images to create 3D images [105–107]. Such 3D analysis has revealed the invasive patterns of tumour cells and the detailed cellular composition of TILs, which have not been elucidated in 2D. Technical breakthroughs are needed to improve the throughput of multiplex 3D tissue imaging, which requires substantial staining and analytical efforts.

## Conclusion

Multiplex imaging has already provided important discoveries and new analytical perspectives in many areas of cancer research, and this method will likely be applied in clinical practice soon. This technique is expected to become a commonly used diagnostic tool in many hospitals as the technology and analytical methods become more standardised. However, antibody-based multicycle imaging techniques, which do not require special equipment or antibody kits, can make tissue-based TME research more flexible and customisable and should be more widely used in the future. In addition, the development of new techniques, including multiomics and 3D, will further expand the capabilities of multiplex imaging in the coming years and will have a major impact on cancer research. Overall, we anticipate that multiplex imaging will further mature technologically and contribute to the diagnosis and treatment of cancer patients through its clinical application in the future.

## Supplementary information

BioRender Publication License
